# Ultrasound-Assisted Extraction of *Spirulina platensis* Carotenoids: Effect of Drying Methods and Performance of the Emerging Biosolvents 2-Methyltetrahydrofuran and Ethyl Lactate

**DOI:** 10.3390/molecules30193881

**Published:** 2025-09-25

**Authors:** Elena Rodríguez-Rodríguez, Ángeles Morón-Ortiz, Paula Mapelli-Brahm, Cassamo U. Mussagy, Fabiane O. Farias, Begoña Olmedilla-Alonso, Antonio J. Meléndez-Martínez

**Affiliations:** 1 Department of Chemistry in Pharmaceutical Sciences, Analytical Chemistry, Faculty of Pharmacy, Complutense University of Madrid (UCM), 28040 Madrid, Spain; elerodri@ucm.es; 2 VALORNUT Research Group (920030-UCM), 28040 Madrid, Spain; 3 Food Colour & Quality Laboratory, Area of Nutrition & Food Science, Universidad de Sevilla, 41012 Seville, Spain; amortiz@us.es (Á.M.-O.); pmapelli@us.es (P.M.-B.); 4 Laboratorio de Desarrollo de Bioprocesos Sostenibles (Labisost), Escuela de Agronomía, Facultad de Ciencias Agronómicas y de los Alimentos, Pontificia Universidad Católica de Valparaíso, Quillota 2260000, Chile; cassamo.mussagy@pucv.cl; 5 Graduate Program of Food Engineering, Department of Chemical Engineering, Polytechnique Center, Federal University of Paraná, Curitiba 80060-000, PR, Brazil; fabianefarias@ufpr.br; 6 Department of Metabolism and Nutrition, Institute of Food Science, Technology and Nutrition (ICTAN-CSIC), 28040 Madrid, Spain

**Keywords:** *Spirulina platensis*, *Arthrospira*, total carotenoids, zeaxanthin, β-carotene, ultrasound assisted extraction, COSMO-SAC, response surface methodology

## Abstract

Extracting bioactives from algae is essential for sustainable solutions aimed at enhancing human health. This study pioneers a multidimensional approach that simultaneously compares ultrasound-assisted carotenoid extraction from spray-dried (SD) and solar-dried (SolD) *Spirulina platensis*, evaluating both food-grade and emerging green biosolvents, validated through COSMO-SAC predictions and optimized using RSM. The SD sample showed higher carotenoid yields with most solvents, consistent with particle size data indicating less aggregation than SolD. Solvent efficacy varied depending on drying method and carotenoid type; acetone was optimal for zeaxanthin and β-carotene from SD and β-carotene from SolD, while methanol and ethanol were more effective for zeaxanthin in SolD. The green solvent 2-methyltetrahydrofuran (2-MeTHF) demonstrated excellent carotenoid affinity in COSMO-SAC predictions and ranked as the second most effective solvent in the SD sample, underscoring its potential as a sustainable alternative. RSM models using 2-MeTHF (SD) and ethanol (SolD) showed excellent prediction accuracy (R^2^ > 98%). Optimized extraction conditions yielded ~4-fold higher total carotenoid recovery compared to non-optimized conditions. Combining computational tools and experiments offers an effective strategy to optimize sustainable extraction of health-promoting carotenoids from *Spirulina*.

## 1. Introduction

Considering the growing population of the planet, the impact of food production on the environment, and the relationship of the diet with disease, there is a global need to produce health-promoting foods in a more sustainable way [1]. The aquatic ecosystems offer many species that can be harnessed in this context, which is aligned with the European Blue Bioeconomy and Biotechnology, aimed at tapping into such biodiversity for commercial exploitation [2]. One example of a success story is the cyanobacteria *Spirulina*, which was already consumed centuries ago by the Aztecs and is currently marketed as a component of health-promoting products. Cyanobacteria are also known as blue-green algae and are photosynthetic microbes related to bacteria. Since they are photosynthetic, it is common to consider cyanobacteria as microalgae, although the former are prokaryotes and the latter are eukaryotes [3]. The production of microalgae offers many advantages in terms of sustainability, including higher yield relative to plants due to their all-year production, ability to grow under stress conditions and in many substrates (including wastewater), easier modulation of biosynthetic pathways, lower nutritional and water needs, and no requirements for herbicides. More than 20 genera of cyanobacteria and microalgae are currently used for food or feed applications, and the numbers are expected to grow considerably [4]. *Spirulina* is a trade name that describes species such as *Arthrospira platensis* and *Arthrospira maxima* and is the most cultivated “microalgae” in the world. It has a Generally Recognised as Safe (GRAS) status by the United States Food and Drug Administration (FDA) and is also approved for human consumption in the European Union. Overall, the importance of *Spirulina* in this context lies in its high protein content (60–70% on a dry weight basis) with high digestibility due to the mucopolysaccharide cell wall, low nucleic acid content, a high total fiber content, and a nutritionally valuable amino acid profile, containing all essential amino acids, similar to the pattern suggested by the FAO [5]. In addition, *Spirulina* contains numerous bioactive compounds, including minerals, γ-linolenic acid, vitamin E, trace elements, B-complex vitamins, and pigments such as chlorophylls, carotenoids, and phycobiliproteins [3,5]. Carotenoids are very versatile compounds that can provide health benefits such as contributing to the reduction in the risk of developing cancers, cardiovascular disease, or eye, skin, neurological, or bone conditions, among others. They are also key in combating vitamin A deficiency, one of the major nutritional concerns in the world, as some of them are precursors of the vitamin. Indeed, they are interesting ingredients of products such as functional foods, supplements, nutraceuticals, or nutricosmetics [6,7].

Drying is a common practice in the food industry, as it leads to the removal of water. This has several advantages, such as weight reduction (with the associated cost reductions in storage or transportation) or the decrease in the enzymatic activity and microbial growth (with the concomitant stability increase). Depending on the technology applied (freeze-drying, solar drying, and spray-drying), products with different characteristics (composition and rheology) are obtained, which can have an impact on aspects as important as bioavailability or extractability [8,9,10]. Spray drying is widely used in the food industry and represents the method of choice for processing microalgal biomass. Owing to the brief exposure time to elevated temperatures, this technique generally yields a stable product of high quality [11]. Regarding solar drying, the sun offers a clean, free, and abundant source of renewable energy, and its use is increasingly considered a practical alternative that reduces dependence on conventional energy. Given fossil fuel scarcity, rising costs, and uncertainties about future availability, solar drying of agricultural products is expected to expand and become more economically viable [12].

The extraction of bioactives from microalgae to be used as ingredients continues attracting much attention. Important topics in this context are the use of intensifying technologies (pressurized liquid extraction, ultrasounds, and microwaves), the assessment of green biosolvents, or the optimization of extraction variables, usually by Response Surface Methodology (RSM) [13,14,15]. In this context, green solvents refer to biodegradable substances with low toxicity for humans and the environment, often derived from renewable resources. Ethyl lactate, for example, is biodegradable and is derived from lactic acid and ethanol, both of biological origin. This solvent holds GRAS (Generally Recognized as Safe) status and is approved by both the FDA and EFSA (European Food Safety Authority) for use as a pharmaceutical ingredient and food additive. It is characterized as non-corrosive, non-carcinogenic, non-teratogenic, biodegradable, and non-ozone-depleting [15]. Similarly, 2-methyltetrahydrofuran (2-MeTHF) is obtained from hemicellulosic biomass and has been recognized for its low toxicity and lower volatility compared to traditional organic solvents [16]. Thus, MeTHF is considered an attractive solvent for industrial use due to its relatively low boiling point (79 °C) and modest enthalpy of vaporization (34 kJ/mol) [15].

Ultrasounds are one of the intensifying technologies that are used for the extraction of carotenoids from diverse matrices. Sonication leads to cavitation, which causes microstructural changes favouring the release of compounds while saving time, solvent, and energy. The ultrasound-assisted extraction of bioactive compounds offers several advantages over traditional methods such as maceration or Soxhlet extraction, including higher extraction efficiency, shorter processing times, lower energy consumption, and increased overall yield [16]. Concerning greener solvents, natural deep eutectic solvents, ionic liquids, and others, such as ethyl lactate or 2-methyltetrahydrofuran (also known as 2-methyl oxolane) are eliciting much interest [15,16]. 2-methyltetrahydrofuran has been recently included in the list of solvents authorized for food use in the European Union [17]. In the ultrasound-assisted extraction (UAE), the solvent is a determining factor, but not the only one. Parameters such as ultrasonic amplitude, time, temperature, or solvent-to-solid ratio affect the intensity of the cavitation process, facilitating cell disruption and the release of compounds such as carotenoids. Therefore, their optimization is essential to achieve efficient and reproducible extraction [18].

Although *S. platensis* has been extensively studied, most studies focus on the recovery of water-soluble proteins such as C-phycocyanin, while carotenoids are usually addressed secondarily, as in the works of Martins et al. [19] and Manisha et al. [20], which are limited to the total pigment yield or the extraction of isolated β-carotene. In contrast, the present study proposes a more targeted approach, combining ultrasound-assisted extraction with probe sonication, response surface methodology, and HPLC analysis to optimize the recovery of key carotenoids such as β-carotene, xanthophyll zeaxanthin, and total carotenoids. Furthermore, two commonly used drying methods in the industry, spray drying and solar drying, are compared to evaluate their influence on carotenoid extractability.

In this context, the objectives of this work were twofold: on the one hand, to assess the performance of traditional food-grade and emerging green solvents for the extraction of carotenoids in spray-dried and solar-dried *Spirulina* biomass; on the other hand, to evaluate the importance of extraction variables and optimize them by response surface methodology.

This study presents a novel integrative approach by simultaneously evaluating the impact of two drying methods (spray and solar) on carotenoid extraction from *S. platensis*, using both conventional and emerging biosolvents (2-MeTHF and ethyl lactate). To our knowledge, this is the first work to combine COSMO-SAC computational modelling with experimental validation and RSM optimization in this context. This multidimensional strategy provides new insights into sustainable extraction processes and solvent–matrix interactions in microalgae.

## 2. Materials and Methods

### 2.1. Reagents and Chemicals

Methyl *tert*-butyl ether (MTBE), (HPLC-grade) was purchased from Honeywell (Seelze, Germany); ethyl lactate from Supelco (Bellefonte, PA, USA); 2-methyloxolane (2-MeTHF) from Sigma-Aldrich (Steinheim, Germany); methanol (HPLC-grade), ethanol, and ethyl acetate (HPLC-grade) from VWR Chemicals (Leuven, Belgium); sodium chloride from PanReac AppliChem (Barcelona, Spain); *n*-hexane (N-Hex), dichloromethane (DCl), petroleum ether (PE) and diethyl ether (DE) were obtained from Análisis Vínicos (Ciudad Real, Spain); acetone (HPLC-grade) from Merck (Darmstadt, Germany).

Lutein (xanthophyll from marigold), zeaxanthin, α- and β-carotene, β-cryptoxanthin, lycopene, potassium hydroxide (KOH), and sodium chloride (NaCl) were obtained from Sigma Aldrich (Madrid, Spain).

### 2.2. S. Platensis Samples

**Figure 1:**
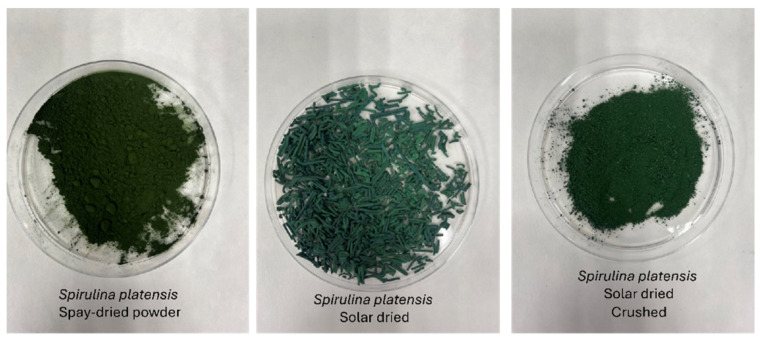
Samples of *S. platensis* dried by spray-drying, solar-dried (original), and solar-dried (crushed).

Organic *S. platensis*, dried by both spray drying (SD) and solar drying (SolD), was acquired in 100 g packages. The materials were produced by the Portuguese company Allmicroalgae (https://www.allmicroalgae.com/en/ (accessed on 21 September 2025)) and provided by Necton (https://necton.pt/ (accessed on 21 September 2025)). According to the supplier’s documentation, the spray drying process involves atomization at inlet air temperatures around 80 °C, while solar drying is performed under controlled air circulation and temperatures below 45 °C. These drying methods aim to preserve the bioactive compounds of *Spirulina*. The SolD *S. platensis* sample was eventually crushed with a mortar and pestle to reduce its particle size (Figure 1).

### 2.3. Particle Size Distribution Study in Solar-Dried and Spray-Dried Spirulina

Particle size analysis was conducted using dynamic light scattering (DLS) with a Zetasizer Pro instrument (Malvern Panalytical; Malvern, UK), and the average particle diameter was accurately calculated using the instrument’s particle size distribution software. The samples SolD and SD were both diluted 1000 times and measured in steady-state conditions.

### 2.4. COSMO-SAC Analysis

The solvent affinities for zeaxanthin and β-carotene were evaluated using Conductor-like Screening Model—Segment Activity Coefficient (COSMO-SAC), a computational model for predicting thermodynamic properties, including molecular interactions based on quantum chemical calculations and statistical thermodynamics. After a proper optimization of the solutes and solvent molecules, the JCOSMO software (version 2.9.15) was applied to predict the solute-solvent affinity through the logarithm of the activity coefficients at infinite dilution (ln γ^∞^) calculation [21,22,23]. In addition, the sigma-profile (σ-profile) of each molecule was considered to elucidate the main interactions between the carotenoids and the evaluated solvents. The ln γ^∞^ value represents how a single solute molecule (zeaxanthin or β-carotene) behaves when completely surrounded by solvent molecules. A lower ln γ^∞^ indicates stronger solute-solvent interactions and higher solubility, whereas a higher ln γ^∞^ suggests weaker interactions, greater deviation from ideality, and reduced solubility of the carotenoid in that solvent.

### 2.5. Carotenoids Extraction

Carotenoid extraction from *S. platensis* was carried out using UAE in two phases: (i) a preliminary solvent screening, and (ii) an optimization study based on RSM. All extractions were performed using a Q500 ultrasonic device (Qsonica, NA, USA) equipped with a 1.6 mm probe operating at a frequency of 20 kHz.

#### 2.5.1. Preliminary Solvent Screening

The recovery of carotenoids from *Spirulina* was performed using solvents with different polarities (methanol, ethanol, acetone, *n*-hexane, DE:PE, ethyl acetate, 2-MeTHF and ethyl lactate) in a fixed solid–liquid ratio to ensure comparability.

For each experiment, 40 mg of *Spirulina* sample (in quadruplicate) was weighed into 15 mL Falcon tubes. In the case of SolD *S. platensis*, the sample was previously ground to a powder using a mortar and pestle to ensure homogeneity. To each sample, 2 mL of the solvent to be tested (methanol, ethanol, acetone, *n*-Hex, DE:PE, ethyl acetate, 2-MeTHF, and ethyl lactate) in a solvent-to-solid ratio of 50 mL/g, was added, followed by vortex agitation at 2500 rpm for 2.5 min. Subsequently, ultrasound was applied using a probe for 2 min at 30% amplitude without pulses. After, samples were centrifuged at 4 °C and 4000 rpm (3220 G) for 5 min to recover the coloured supernatant. This extraction and centrifugation process was repeated a minimum of three times until the colour completely disappeared. The combined supernatants were dried in a rotary evaporator, and the dry extracts were stored at −20 °C under a nitrogen atmosphere in polypropylene microcentrifuge tubes with blue screw caps. For extracts obtained with ethyl lactate, a washing step was performed prior to evaporation: 3 mL of dichloromethane and 4 mL of distilled water were added to 6 mL of extract, manually shaken (30 s), and centrifuged (2 min, 4 °C, 3220 G), removing the clear aqueous phase. This process was repeated until 2–3 mL of coloured extract was obtained, concluding with an additional wash with 5% NaCl.

The extraction conditions employed (ratio 50:1 (*v*/*m*) in this study were selected based on previous research that demonstrated their effectiveness in recovering bioactive compounds from algal matrices. This ratio facilitates optimal solubilization and enhances mass transfer, thereby improving extraction efficiency without causing system saturation [24]. The sonication parameters were chosen to prevent thermal degradation of sensitive metabolites such as pigments and antioxidants, following strategies reported in studies using *Chlorella sorokiniana* and *Dunaliella bardawil*, where similar conditions—including prior grinding and green solvents—were successfully applied [15,25].

The quantification of total carotenoids was performed spectrophotometrically using a diode array spectrophotometer (model G1103B, Agilent Technologies, Santa Clara, CA, USA), following the Lambert-Beer law, which relates absorbance to concentration, path length, and the specific absorption coefficient of the compound of interest. This law ensures that absorbance measurements are reliable only within the linear range of the method. The dry extracts were reconstituted in 4 mL of petroleum ether and appropriate dilutions (1:2, 1:5 or 1:10) were prepared when necessary. Total carotenoids were calculated according to the following equation: C (mg/mL) = (A × 1000)/(E × l), where A is the absorbance of the extract at the selected wavelength (450 nm for carotenoids), E is the specific absorption coefficient ($E_{cm}^{\%}$ = 2592 for β-carotene in petroleum ether), and l is the path length of the cuvette (1 cm). The total carotenoid content was expressed as β-carotene equivalents.

After measurement, the extracts were re-dried in a rotary evaporator and stored at −20 °C under nitrogen for subsequent HPLC analysis.

#### 2.5.2. RSM Study

To further investigate and optimize the extraction process, a central composite design (CCD) with three independent variables was applied. Considering the quantification of the two main carotenoids (zeaxanthin and β-carotene), the RSM study was conducted using ethanol for the SolD matrix (a green solvent and the most efficient extractor) and 2-MeTHF for the SD matrix (the second most efficient extractor, also classified as green). The variables studied were ultrasound amplitude (A, %), extraction time (B, min) and solvent-solid ratio (C, mL/g). Each factor was tested at five levels: low (−1), centre (0), high (+1), and two axial levels. The independent parameters and their corresponding low, middle, and high coded levels were as follows: amplitude (A) at 30%, 50%, and 70%; extraction time (B) at 1, 2, and 4 min; and solvent-to-solid ratio (C) at 10, 30, and 50 mL/g. The parameters selected for optimization were chosen based on previous studies conducted in our laboratory [15]. The responses evaluated were zeaxanthin, β-carotene, and TCC.

The objective was to evaluate the influence of these variables on the recovery of total carotenoid content (TCC) and specific carotenoids, including zeaxanthin and β-carotene, and to determine the optimal extraction conditions. The experimental design was based on a second-order polynomial regression model and included statistical tools such as analysis of variance (ANOVA), as well as the generation of contour and surface graphs using Stat-Ease 360^®^ software (version 23.1.7, 64-bit, trial version; Stat-Ease Inc., Minneapolis, MN, USA).

The adequacy of the model was evaluated using the coefficient of determination (R^2^), the adjusted R^2^, and the sum of squares of prediction error (PRESS). Table 1 presents the complete CCD, which included 20 experimental trials and six replicates at the centre point to ensure reproducibility.

The general form of the adjusted regression model is shown in Equation (1):

(1) $$Y=\beta 0+\sum_{i=1}^{k}\beta ixi+\sum \sum_{i<j}\beta ijxixj+\sum_{i=1}^{k}\beta iix_{i}^{2}+\epsilon$$

In this equation, *Y* is the predicted response (e.g., carotenoid content); *β_0_* is the intercept; *βi*, *βij*, and *βii* are the coefficients for the linear, interaction, and quadratic terms, respectively; *xi* and *xj* are the coded independent variables, namely amplitude (%), extraction time (min), and solvent-to-solid ratio (mL/g); and *ε* is the residual error, which is assumed to have a mean of zero.

The samples were weighed precisely to adjust to different solvent-to-solid ratios: 10, 18.1, 30, 41.9, and 50 mL/g, as indicated in Table 1. In each case, 5 mL of the best green extraction solvent was systematically used as the extraction solvent. The experimental series was performed with different amplitudes (30%, 38.1%, 50%, 61.9% and 70%) and extraction times (1, 1.61, 2.5, 3.39 and 4 min), according to the experimental design described in Table 1.

To protect the carotenoids from thermal degradation, the samples were placed in an ice bath throughout the sonication process. After each extraction step, the samples were centrifuged to separate the liquid phase. This extraction cycle was repeated until the solid residue appeared colourless, indicating complete removal of the pigment.

The supernatants collected in each cycle were pooled and concentrated using a rotary evaporator under reduced pressure, maintaining the water bath temperature below 30 °C. The concentrated extracts were then stored at −20 °C in a nitrogen atmosphere until subjected to chromatographic analysis.

### 2.6. High-Performance Liquid Chromatography (HPLC) Analysis

Carotenoids, isolated according to the procedure detailed in Section 2.5, were quantified using High-Performance Liquid Chromatography (HPLC). The HPLC system comprised a Waters Alliance e2695 separation module, a Waters 2998 photodiode array (PDA) detector (Waters, Milford, MA, USA), and a YMC C30 column (5 µm, 250 × 4.6 mm i.d.) with a guard column (Aquapore ODS type RP-18). The analysis was conducted at ambient temperature (approximately 25 °C). The mobile phase consisted of a gradient mixture of methanol containing 0.1% trimethylamine (solvent A) and MTBE (solvent B). The gradient program was as follows: initial conditions of 95% solvent A and 5% solvent B, transitioning to 70% A and 30% B at 25 min, then to 35% A and 65% B at 55 min, and finally returning to 95% A and 5% B at 60 min. Detection of carotenoids was performed at 450 nm. Empower 2 software, (Waters, Milford, MA, USA) was used for chromatogram processing [26]. Carotenoids were identified by comparing their retention times and absorption spectra to those of known standards. Calibration curves were generated using commercial carotenoid standards, with a range of 0.28–4.76 ng/μL for zeaxanthin and 0.8–12.8 ng/μL for β-carotene.

The experimental design described in Table 1 was carried out using a high-performance liquid chromatography system (1260 Infinity II Prime LC, Agilent Technologies) equipped with a diode array detector (DAD) and a C_30_ column (YMC, 150 × 4.6 mm, particle size 3 µm). Before injection, the dried extracts were reconstituted in approximately 200 µL of ethyl acetate, and 10 µL aliquots were introduced into the system. The mobile phase consisted of methanol, MTBE, and water, applied in linear gradient mode. The flow rate was set at 1 mL/min. This chromatographic method followed a previously validated protocol developed by our group [27]. The quantification of individual carotenoids was performed using external calibration curves prepared with pure standard compounds, as described by Stinco et al. [27]. These two experimental phases were carried out in different laboratories, each using its standard HPLC protocol and validated according to the available instrumentation. Although the methods differ slightly in chromatographic conditions, both were independently validated and are suitable for carotenoid quantification.

### 2.7. Statistical Study

Results for quantitative variables are expressed as mean and standard deviation (Std. Dev.). The Shapiro–Wilk test was performed to check for normality. β-carotene followed a non-normal distribution, while total carotenoids measured by spectrophotometer and zeaxanthin conformed to a normal distribution.

To assess differences in the extraction capacity of each solvent when comparing SolD and SD *S. platensis*, the Mann–Whitney U test was used for the former and Student’s *t*-test for the latter. To compare the extraction efficiency of the different solvents within each *S. platensis* group (SolD or SD), a one-way ANOVA with Tukey’s post hoc tests was applied when data followed a normal distribution. For non-normal distributions, the non-parametric Kruskal–Wallis test was used, followed by paired comparisons with the Mann–Whitney U test and Bonferroni correction.

Values were evaluated at the 5% significance level using two-sided tests. IBM SPSS Statistics 25.0 software was used for the statistical analysis.

## 3. Results and Discussion

### 3.1. Particle Size Distribution Study in Solar-Dried and Spray-Dried Spirulina

**Figure 2:**
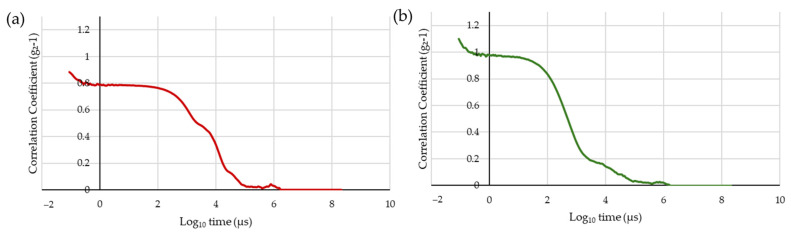
Autocorrelation function of samples SolD (**a**) and SD (**b**) measured by Dynamic Light Scattering (DLS), illustrating the signal fluctuation over time (µs).

A relatively homogeneous particle distribution is observed, as depicted in Figure S1, where a single and well-defined peak is observed. SolD (Figure S1a) revealed a peak located near 267 nm, whereas for SD (Figure S1b), the peak appears around 230 nm. Figure 2a,b, represents the autocorrelation function of the DLS analysis, which measures signal fluctuations over time (µs). In this test, a smooth and well-defined decay indicates good sample dispersion. In SolD (Figure 2a), more noise is detected, confirming the presence of large aggregates. In SD (Figure 2b), the decay is cleaner, suggesting that aggregates were more effectively reduced. Both samples exhibit particles predominantly in the 230–267 nm range. SolD displays higher interference due to fluorescence, requiring the use of optical filters to obtain more accurate measurements. SD appears to have fewer aggregates compared to SolD, as reflected by a lower Z-average (456 nm vs. 2851 nm in SolD).

The observed differences in particle size and aggregation for *S. platensis* processed by SolD and spray drying (SD) are consistent with the known effects of these drying technologies on heat-sensitive biomaterials. The smaller particle size and reduced aggregation observed with spray drying (SD) can be attributed to the rapid evaporation and solidification of droplets in the hot air stream, which minimizes the time available for particle agglomeration to occur [28]. This is in stark contrast to the SolD process, where a noisy DLS autocorrelation function and a significantly higher Z-average (2851 nm versus 456 nm for SD) indicate a broad particle size distribution and the presence of substantial aggregates. This phenomenon of aggregation during drying is a common challenge for food powders and protein isolates, often leading to poor solubility and reduced functionality [29]. The high Z-average value in the SolD sample, in particular, is a clear indicator of the intensity-weighted mean particle size, which is highly sensitive to the presence of large particles or aggregates and thus provides strong evidence for a more polydisperse sample [30]. These findings align with previous studies on food matrices such as mango, carrot, and tomato, where spray drying consistently resulted in better retention of carotenoids, improved powder quality, and reduced aggregation compared to sun drying. For instance, in fruits and vegetables, spray drying yielded finer particles with superior nutritional and physical properties, whereas sun drying led to greater degradation and heterogeneity in particle size [31,32].

### 3.2. Results from the COSMO-SAC Study

**Figure 3:**
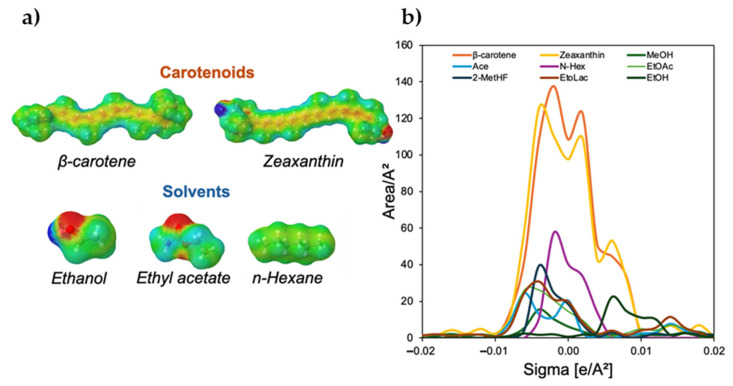
(**a**) 3D induced surface charge densities; (**b**) σ-profiles of zeaxanthin and β-carotene and some selected solvents investigated in this work generated by COSMO-SAC. Ace: acetone; 2-MeTHF: 2-methyloxolane; N-Hex: *n*-hexane; EtOLac: ethyl lactate; MeOH: methanol; EtOAc: ethyl acetate; EtOH: ethanol.

In Figure 3a, the 3D-induced surface charge densities of carotenoids and some representative solvents investigated in this work generated by COSMO-SAC are shown. In the molecules, the red and blue areas around the molecular structures represent induced charge distributions, corresponding to regions of positive (hydrogen bond acceptor) and negative (hydrogen bond donor) charges, respectively. In contrast, the green regions indicate neutral or nonpolar areas of the molecules.

Figure 3b presents the σ-profile of zeaxanthin (yellow), β-carotene (orange), and various solvents (different colours), illustrating their charge distribution. Both carotenoids predominantly exhibit neutral charge density regions (between −0.01 and 0.01 e/Å^2^), with only minor polar contributions beyond this range for zeaxanthin. The coexistence of polar and nonpolar regions in zeaxanthin suggests that their interactions with solvents involve a combination of hydrophobic forces and specific polar interactions, such as hydrogen bonding, particularly in solvents with higher polarity. β-carotene, which exhibits only a nonpolar profile, meaning it interacts best with nonpolar solvents such as *n*-Hexane (purple line) or ethyl acetate (green line). Its limited polar regions suggest weak hydrogen bonding interactions, making it poorly soluble in highly polar solvents like methanol (dark green). Zeaxanthin shows a more balanced distribution between nonpolar and polar regions, due to the presence of hydroxyl (-OH) groups in its structure, zeaxanthin can establish hydrogen bonds, leading to better solubility in moderately polar solvents like ethyl acetate or even acetone, while it still interacts well with nonpolar solvents, its slight polarity enables additional affinity with polar-aprotic solvents like 2-MeTHF (dark blue) and ethyl lactate (brown).

**Figure 4:**
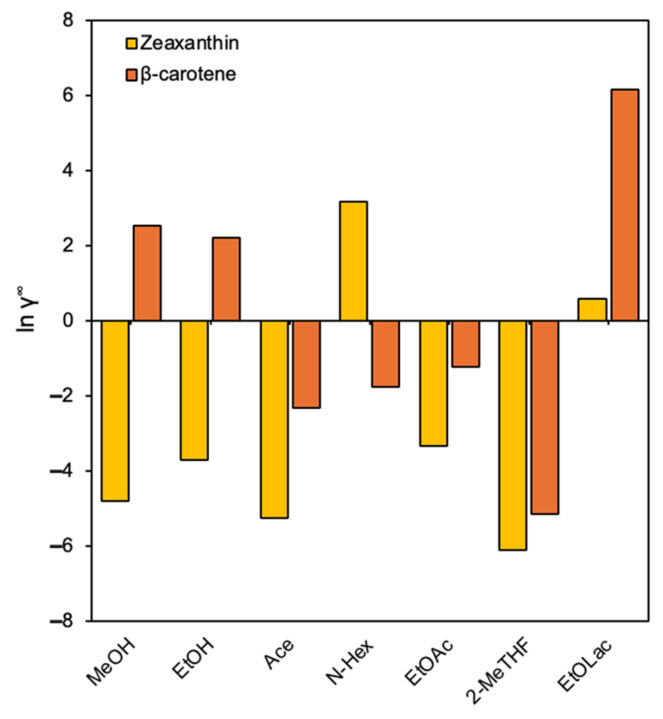
Predicted activity coefficient in infinite dilution (ln ϒ∞) of zeaxanthin (yellow bars) and β-carotene (orange bars) in selected solvents using COSMO-SAC. Ace: Acetone; 2-MeTHF: 2-methyloxolane; N-Hex: *n*-Hexane; EtOLac: Ethyl Lactate; MeOH: Methanol; EtOAc: Ethyl acetate; EtOH: Ethanol.

In addition, through the ln ϒ∞ calculation, as shown in Figure 4, it is possible to classify the solvents according to their affinity with each solute. The solvent affinity trend for zeaxanthin followed the order: 2-MeTHF ~ Acetone ~ Methanol > Ethanol > Ethyl acetate > Ethyl lactate > *n*-Hexane, while for β-carotene it was 2-MeTHF ~ Acetone ~ Ethyl acetate ~ *n*-Hexane > Ethanol > Methanol > Ethyl lactate. Both carotenoids exhibited strong affinity for 2-MeTHF and acetone, highlighting their potential as promising solvents for extraction. Zeaxanthin demonstrated a preference for polar solvents (alcohols, ketones), likely due to its hydroxyl (-OH) functional groups, which enable hydrogen bonding. In contrast, β-carotene favoured nonpolar to moderately polar solvents, consistent with its hydrophobic nature. Note that ethyl lactate was unsuitable for β-carotene, while *n*-Hexane was ineffective for zeaxanthin (Figure 4).

With respect to hexane, in a study in which the COSMO-SAC model was applied to assess the thermodynamic interactions between carotenoids and selected solvents, providing insight into their extraction efficiency, the results showed that hexane was not the best option for carotenoid recovery, as indicated by its positive infinite dilution activity coefficient (IDAC) values for xanthophylls. These values suggest a low affinity between hexane and these more polar carotenoids, which correlates with the lower experimental extraction yields observed. In contrast, acetone exhibited the best extraction performance, displaying the lowest IDAC values across all analysed carotenoids. Although hexane has traditionally been favoured for carotenoid extraction [33], its limitations in extracting xanthophylls suggest a need for alternative solvents that offer higher efficiency and broader applicability in microalgal biorefineries [34]. Regarding ethyl lactate, despite being considered a “green” solvent and thus offering environmental advantages [16,35], it appears to be less efficient for carotenoid extraction under these conditions. This could be due to its specific polarity or its limited ability to penetrate the algae matrix and solubilize the carotenoids.

### 3.3. Total Carotenoids Extracted as a Function of the Drying Method and the Solvent

In the current study, the range of total carotenoids measured by spectrophotometry (178 to 2013 µg/g) in *S. platensis* is lower than that found in *Arthrospira platensis* (as *Spirulina* is currently called) from Korea after extraction with acetone (280 to 4430 µg/g dry weight) [36]. It is also lower than what was observed in an *S. platensis* sample from Italy, where the carotenoid concentration obtained after extraction with 96% ethanol was 0.5 mg/g dry weight [37]. However, it is similar to the values described in dietary supplements purchased in Czech Republic pharmacies, which contained only pure bio-quality dried *S.* with no additional excipients, following acetone extraction (1420 and the lowest 230 µg/g) [38]. The variability found across different studies can be influenced by various factors. Thus, the biosynthesis of carotenoids in microalgae and other microbes is easily modifiable by the growing conditions and is sometimes much modified by conditions such as salt concentration, temperature, nutrient reduction or high-intensity light exposure, among others. Other factors explaining such a difference can be post-harvest storage conditions (like drying), as well as the extraction method and solvent [4,16]. In the UAE, factors such as extraction amplitude, time, temperature, pH, and solvent-to-solid ratio have been shown to significantly influence the recovery of pigments and other bioactive compounds [39,40].

**Figure 5:**
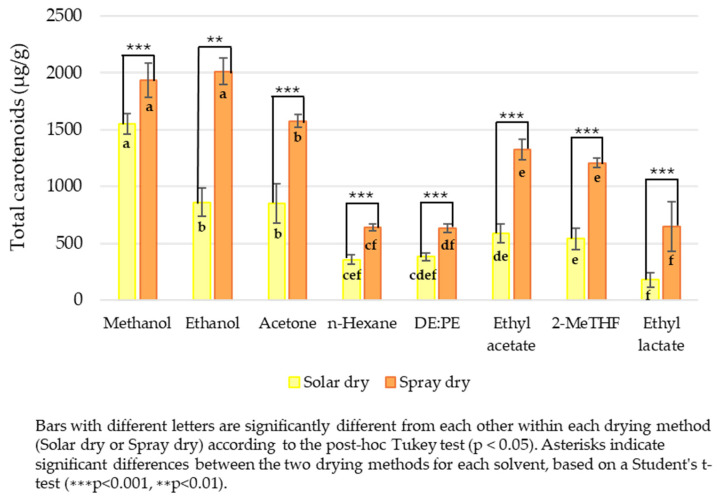
Total carotenoids determined spectrophotometrically and expressed as β-carotene equivalents (μg/g dry weight): Differences between drying methods and solvents.

Regarding the drying method, it has been described that both solar and hot-air-based drying processes, commonly used in the mass production of most commercial *Spirulina* powders, could decrease the pigment content of the powder [36]. Therefore, the algae’s pre-drying method would significantly impact carotenoid extraction efficiency. In line with this, SD *S. platensis* showed higher carotenoid extraction yields for most solvents used—634.58 µg/g (DE:PE) and 2013.06 µg/g (ethanol)—compared to SolD *S. platensis*, which ranged from 178.23 µg/g (ethyl lactate) to 1548.64 µg/g (methanol) (Figure 5). This could be attributed to several factors: spray drying is a rapid process that minimizes exposure to high temperatures and oxygen, which reduces the degradation of carotenoids, compounds highly sensitive to oxidation and light. Additionally, spray drying leads to microencapsulation and alteration of the cellular matrix, which can protect these compounds and facilitate their release during extraction. In contrast, solar drying, while more economical, can involve prolonged exposure to UV light, high temperatures, and oxygen. This leads to greater degradation through oxidation and geometrical isomerization (*trans* to *cis*), as well as compaction of the matrix that hinders solvent diffusion, resulting in lower extraction yields. In addition, particle size plays a crucial role in mass transfer during solid–liquid extraction, directly influencing the effective recovery of carotenoids from *Spirulina* biomass. For example, larger or more aggregated particles reduce the surface area available for solvent interaction, leading to lower extraction efficiency, while smaller, more uniform particles with reduced aggregation enhance mass transfer and allow for better solvent penetration and consequently high solubilization of intracellular biomolecules. These factors contribute to explaining why SD exhibited higher extraction yields (Table 2, Figure 5), as its reduced particle size (Figure 1 and Figure 2) and improved dispersion facilitated a more efficient mass transfer, enhancing the recovery of zeaxanthin and β-carotene from intracellular biomass.

Generally, ethanol and methanol showed the highest extraction power across both drying methods. For SolD *S. platensis*, the most effective solvents were methanol (1548.64 µg/g), followed by ethanol (861.04 µg/g) and acetone (848.83 µg/g), while n-hexane (356.83 µg/g), DE:PE (382.35 µg/g) and ethyl lactate (178.23 µg/g) were the least effective. In SD *S. platensis*, ethanol (2013.06 µg/g) and methanol (1933.67 µg/g) yielded the highest extraction, with ethyl acetate (1325.88 µg/g) also showing good extractive capacity; conversely, *n*-Hexane (641.23 µg/g), DE:PE (634.58 µg/g), and ethyl lactate (647.23 µg/g) were the least effective solvents for this group (Figure 5). This is consistent with the nature of many carotenoids, which are fat-soluble pigments but can also possess hydroxyl groups, making them soluble in polar or intermediate-polarity solvents like alcohols. The robustness of their performance positions them as priority options in the development of extraction processes. Similar to our results, in a study comparing different solvents for extracting chlorophyll *a* and carotenoids from *S. platensis*, the results indicated that methanol was the most efficient solvent for extracting plant pigments, followed by ethanol and acetone [41]. Other intermediate-polarity solvents, such as acetone, 2-MeTHF, and ethyl acetate, also demonstrated considerable extraction capacity, though generally lower than that of ethanol and methanol. Their effectiveness can be attributed to their ability to dissolve compounds with diverse polarity ranges, including various types of carotenoids. Their use could be considered based on other criteria such as toxicity, ease of recovery, or cost. In this regard, 2-MeTHF presents several favourable characteristics: low toxicity when ingested, it is non-toxic through dermal exposure or inhalation, exhibits negative genotoxicity, and is not mutagenic. Although it can lead to severe eye damage and skin irritation, it is not categorized as a skin sensitizer. Furthermore, its application in pharmaceutical chemical processes has received approval [42]. Conversely, solvents like hexane, DE:PE, and ethyl lactate showed the lowest carotenoid extraction yields. The low efficiency of hexane and DE:PE, both non-polar in nature, suggests that the carotenoids present in this alga are predominantly polar or are in matrices that hinder their extraction by non-polar compounds. The successful application of 2-MeTHF as a highly effective green biosolvent for *Spirulina* carotenoids, particularly for spray-dried samples, significantly expands the scope of sustainable extraction practices. This finding, reinforced by our COSMO-SAC predictions, provides strong evidence for 2-MeTHF’s potential as a viable and environmentally benign alternative to traditional solvents, a contribution not previously demonstrated with such comprehensive validation in this matrix.

Thus, considering the obtained results, it is observed that solvent polarity plays a crucial role, with polar solvents like ethanol and methanol being the most versatile and effective. This is consistent with other studies conducted on microalgae, specifically *Dunaliella bardawil* [25] and *Chlorella sorokiniana* [15], in which these solvents also proved to be among the most effective for carotenoid extraction.

Additionally, recent studies have demonstrated that the combination of ultrasound-assisted extraction (UAE) with green solvents represents an effective and sustainable alternative to traditional carotenoid extraction techniques. For instance, Drosaki et al. [43] extracted 0.672 mg/100 g dry peels of total carotenoids from peach peels using vegetable oils and ultrasound, achieving yields comparable to conventional hexane (0.672 mg/100 g dry peels) but with reduced environmental impact. Similarly, Martínez-Girón et al. [44] reported 151.5 mg/100 g of total carotenoids from peach palm peel using UAE with soybean oil, surpassing traditional maceration by According to IUPAC recommendations, the stereochemical descriptors *cis* and *trans* should be written in italics and lowercase when used in compound names.33.6%. In the case of tomato by-products, Mozafari et al. [45] obtained 2586 mg/kg of lycopene and 161 mg/kg of β-carotene through UAE with ethanol. Focusing on microalgae, Morón-Ortiz et al. [15] employed UAE combined with green solvents such as 2-methyltetrahydrofuran (2-MeTHF) and ethyl lactate to extract total carotenoids from *Chlorella sorokiniana*. Thanks to a milling pretreatment, they achieved yields as high as 5546.96 µg/g, reinforcing the efficiency of UAE with alternative solvents. Altogether, these findings highlight that green solvents—such as vegetable oils, alcohols, and cyclic ethers—when combined with ultrasound not only improve extraction efficiency but also reduce processing time, energy consumption, and compound degradation, positioning UAE as a promising alternative to conventional techniques like Soxhlet or hexane-based maceration.

The findings from this analysis are particularly relevant for the initial selection of solvents in carotenoid extraction processes, suggesting a focus on those with higher solubilization capacity to maximize yield. However, it is important to consider the specific interaction of the solvent with the algae matrix (which can be altered by drying), as this remains an underlying factor influencing extraction performance.

### 3.4. Carotenes and Xanthophylls Extracted as a Function of the Drying Method and the Solvent

**Figure 6:**
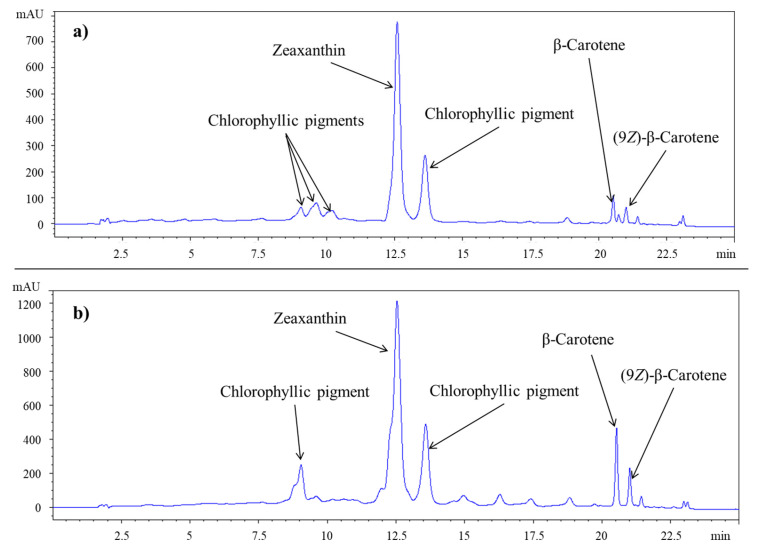
Chromatograms at 450 nm of a sample of SolD *S. platensis* extracted with ethanol (**a**) and SD *S. platensis* extracted with 2-MeTHF (**b**) using the optimized conditions in each case.

According to the HPLC analysis, β-carotene and zeaxanthin were identified as the major carotene and xanthophyll, respectively (Figure 6), which aligns with other studies. For instance, in a study conducted on two different strains of *S. platensis* cultivated in Egypt, β-carotene was identified as the major carotenoid [46], with β-cryptoxanthin, zeaxanthin, canthaxanthin, antheraxanthin, echinenone, and fucoxanthinol also being found. Similarly, the major carotenoids identified in *Arthrospira platensis* from Korea [36] included all-*trans*-β-carotene, all-*trans*-zeaxanthin, 9-*cis*-β-carotene, 13-*cis*-β-carotene, and diatoxanthin. Among them, the content in all-*trans*-β-carotene (0.02 to 2.30 mg/g) and all-*trans*-zeaxanthin (0.09 to 1.27 mg/g) stood out. In another study, conducted on commercial products containing *S. platensis*, analysis showed a content of β-carotene in a range from 86.85 µg/g to 1037.07 µg/g) and zeaxanthin from 27.74 µg/g to 606.36 µg/g [38].

**Figure 7:**
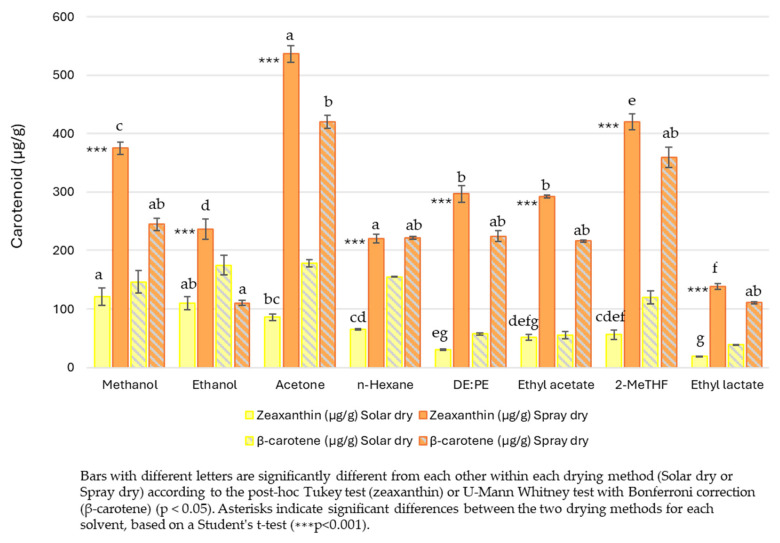
Zeaxanthin and β-carotene extracted with each solvent in *S. platensis* dried by solar (yellow bars) or spray dry (orange bars).

However, the results revealed a different proportion of these compounds depending on the drying method. For SolD *S. platensis*, a higher concentration of β-carotene was observed compared to zeaxanthin. Conversely, SD *S. platensis* showed the opposite pattern, with a greater extraction of zeaxanthin. Regardless of the specific solvent used, SD *S. platensis* consistently yielded a higher amount of both carotenoids compared to SolD *S. platensis*. An exception was the extraction of β-carotene with ethanol (Figure 7). This highlights the importance of considering the individual stability of each carotenoid and its interactions with the matrix when performing their extraction. This difference in carotenoid proportion suggests that the spray-drying method might be more efficient in preserving or extracting zeaxanthin, that β-carotene could be more susceptible to degradation under these conditions, or that the cellular matrices are altered differently by each drying type, affecting the extractability of each carotenoid [47].

Our distinct finding regarding the differential impact of spray-drying versus solar-drying on zeaxanthin and β-carotene extractability (Figure 7) represents a novel insight into *Spirulina* processing, suggesting that drying methods not only affect overall yield but also the relative proportions of specific carotenoids, which is a critical consideration for tailored applications.

The influence of drying methods and solvent selection on carotenoid extraction has been previously explored in various matrices. Drying not only reduces moisture content and facilitates solvent penetration but also alters the physical and chemical structure of the matrix, which can significantly affect carotenoid stability and extractability. For example, Gandul-Rojas et al. [48] examined olive fruits and found that dimethylformamide was effective for extracting chlorophylls and monoesterified xanthophylls, while hexane was more suitable for nonpolar carotenoids like β-carotene. However, the drying conditions prior to extraction were critical: high-temperature drying can degrade sensitive pigments, while milder techniques help preserve them. Similarly, Rebecca et al. [49] compared carotenoid yields from vegetables such as carrot, spinach, and capsicum using hexane/acetone (1:1) and ethanol, noting that carrots yielded the highest β-carotene content. The study emphasized that solvent polarity significantly influenced extraction efficiency, but also that drying temperature and duration affected pigment retention. In Brassica vegetables, Podsędek et al. [50] used UPLC to show that drying and solvent combinations affected the recovery of both carotenes and xanthophylls. Methanol-based extractions provided better resolution and quantification of lutein and β-carotene, especially when freeze-drying or low-temperature oven drying was applied. Freeze-drying, in particular, was found to preserve carotenoid integrity by minimizing thermal degradation and oxidation. Recent studies have also explored innovative drying techniques such as infrared-assisted drying, vacuum drying, and non-thermal air-drying. For instance, Lazzarini et al. [10] demonstrated that non-thermal air-drying of tomato pomace preserved lycopene and β-carotene more effectively than conventional heat drying, especially when combined with green solvents like ethyl lactate.

Regarding solvent efficacy, it varied not only based on the drying method applied but also on the type of carotenoid extracted. In the case of zeaxanthin, polar alcohols like methanol (120.75 µg/g) and ethanol (110.01 µg/g) were most effective in SolD *S. platensis* (F (7, 10) = 39.119, *p* < 0.001). However, in the SD *S. platensis* matrix, acetone (536.74 µg/g) and 2-MeTHF (420.04 µg/g) demonstrated superior extractive capacity (Figure 7). This difference could be attributed to changes in matrix polarity or the exposure of new binding sites post-spray drying, allowing for more efficient solubilization by these solvents. In this regard, acetone also proved to be among the most efficient solvents to extract violaxanthin, lutein, zeaxanthin, and carotene from two microalgae, *Chlorella sorokiniana* and Heterochlorella luteoviridis, in comparison with ethanol, ethyl acetate, and hexane [34]. It is worth noting that the ability of 2-MeTHF to match or surpass conventional solvents in carotenoid extraction is a promising finding for the development of more sustainable processes, as it is considered a green solvent that can be obtained from carbohydrate feedstocks and exhibits lower toxicity as compared to petroleum-derived hexane [42].

In the case of β-carotene, although the overall Kruskal–Wallis test indicated significant differences in the extractive power of the solvents used in SolD *S. platensis* (test statistic = 15.550, df = 7, *p* = 0.030) and SD *S. platensis* (test statistic = 18.367, df = 7, *p* = 0.010), post hoc pairwise comparisons with Bonferroni correction did not reveal statistically significant differences among most solvent pairs. (Figure 7). This suggests that β-carotene, being less polar than zeaxanthin, might require more specific extraction conditions or face greater barriers to release from the matrix.

On the other hand, ethyl lactate, DE:PE, and hexane showed the lowest efficiency for both carotenoids under both drying conditions (Figure 7). This indicates that they are not the optimal solvents for the extraction of specific carotenoids from this matrix, suggesting unfavourable polarity or interaction properties with the algae’s structure, as previously mentioned.

Thus, the experimental results (Figure 7) align with the COSMO-SAC predictions (Figure 3) and these findings reinforce the importance of selecting solvents based on the polarity and functional characteristics of the target carotenoid to optimize solubility and extraction efficiency. Computational tools like COSMO-SAC provide a good theoretical basis for understanding why certain solvents perform better, especially by highlighting solvent-carotenoid interactions (e.g., the preference of zeaxanthin for polar solvents and β-carotene for nonpolar to moderately polar solvents). At this point, it is important to note that the direct extrapolation of IDAC results is not always possible, as carotenoids inside matrices are not isolated; rather, they are interacting with structures and are in the presence of other compounds.

### 3.5. Results from the RSM Study

The UAE of carotenoids from *S. platensis* matrices (SD and SolD) was successfully optimized using RSM. The developed models of extraction of carotenoids from SD and SolD *S. platensis* matrices demonstrated excellent predictive capability for individuals (zeaxanthin and β-carotene) and TCC, with R^2^ values consistently above 98%. The lack-of-fit tests (*p* > 0.05) confirmed the robustness of the models, and ANOVA results validated their statistical significance (*p* < 0.001), highlighting the appropriateness of the selected variables (amplitude (%), time (min), and solvent-to-solid ratio (mL/g)) for optimizing the process [51]. The RSM study was conducted using ethanol for the SolD matrix (a green solvent and the most efficient extractor) and 2-MeTHF for the SD matrix (the second most efficient extractor, also classified as green). The parameters selected for optimization (ultrasound amplitude (30–70%), extraction time (1–4 min), and solvent-to-solid ratio (10–50 mL/g)) were chosen based on previous studies conducted in our laboratory [15]. The responses evaluated were zeaxanthin, β-carotene, and TCC.

#### 3.5.1. Spray-Dried *S. platensis*

In the SD matrix, the model showed excellent predictive ability with R-squared values exceeding 98% for zeaxanthin, β-carotene, and TCC. The ANOVA confirmed the significance of the three models (zeaxanthin, β-carotene, and TCC) (*p* < 0.001), and lack-of-fit was not significant (*p* > 0.05) (Table S1), indicating adequate model fitting.

**Figure 8:**
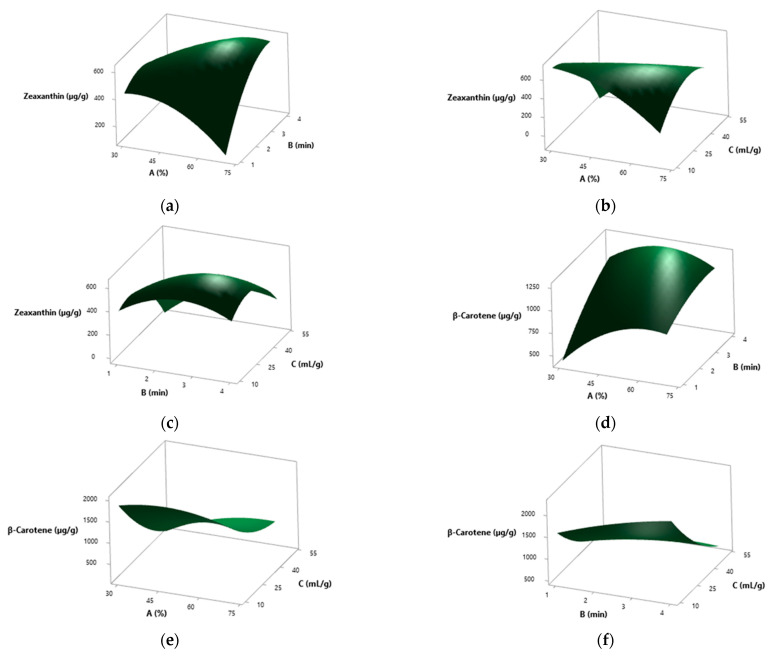

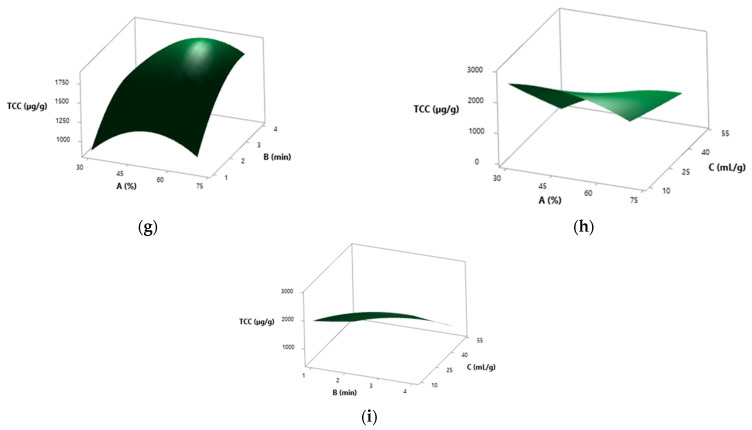
Three-dimensional surface plots showing the interactions between factors in the ultrasound-assisted extraction of zeaxanthin, β-carotene, and total carotenoid content (TCC) from spray-dried *S. platensis*. (**a**–**c**) Interactions for zeaxanthin: (**a**) A vs. B at constant C = 30 mL/g; (**b**) A vs. C at constant B = 2.5 min; (**c**) B vs. C at constant A = 50%; (**d**–**f**) Interactions for β-carotene: (**d**) A vs. B at constant C = 30 mL/g; (**e**) A vs. C at constant B = 2.5 min; (**f**) B vs. C at constant A = 50%; (**g**–**i**) Interactions for total carotenoid content (TCC): (**g**) A vs. B at constant C = 30 mL/g; (**h**) A vs. C at constant B = 2.5 min; (**i**) B vs. C at constant A = 50%. Here, A = Amplitude (%); B = Time (min); C = Solvent-to-solid ratio (mL/g).

In the case of zeaxanthin, the solvent-to-solid ratio emerged as the most relevant variable, closely followed by extraction time (*p* < 0.001) (Table S1). Although amplitude did not display a significant effect (*p* = 0.816), it was involved in notable quadratic and interaction effects. All tested 2-way interactions evaluated (amplitude × amplitude, amplitude × time, time × solvent-to-solid ratio) resulted in highly significant (*p* < 0.001) (Table S1). For a better understanding of the interactions between factors influencing zeaxanthin extraction, the corresponding 3D surface plots are shown in Figure 8a–c.

These plots illustrate how amplitude, time, and solvent-to-solid ratio jointly affect the extraction yield. The lowest zeaxanthin recovery was obtained in run 8 (38.1%, 1.6 min, 41.9 mL/g), yielding 258.20 µg/g, whereas the best-performing condition was run 1 (38.1%, 1.6 min, 18.1 mL/g), yielding 670.24 µg/g (Table 2). The model predicted maximum extraction at 30% amplitude, 1.79 min, and 10 mL/g, corresponding to 747.50 µg/g. Experimental validation produced a slightly higher yield of 780.39 µg/g.

The regression equation for individual and TCC in the SD matrix, expressed in terms of coded variables, along with the corresponding coefficients of determination (R^2^), is presented in Table 3.

These findings are consistent with previous studies. Wang et al. [52] optimized the extraction of zeaxanthin and lutein from dried corn gluten meal using ultrasound-assisted extraction. They observed higher extraction efficiency under mild ultrasound conditions (40 kHz, 250 W, 7 mL/g, 45 min (within the 30–50 min range)), which helped to avoid excessive temperatures (above 56 °C), minimizing carotenoid degradation. All individual and interaction effects were significant (*p* ≤ 0.001). Similarly, Janepinich et al. [53] found that amplitudes >33% degraded lutein in marigold flower extraction (750 W, 15 mL/g), identifying 32.76% as optimal. Time, also significant in both *S. platensis* matrices, was the most influential factor in marigold flowers; however, no significant interactions were found.

Additionally, the optimization of the UAE of zeaxanthin from freeze-dried *Dunaliella tertiolecta* using methanol: dichloromethane (3:1) showed optimal conditions at 33.6 min (9.6–110.4 min), 59.2 °C (6.4–73.6 °C), and 1:62 (g/mL) (1:11.64–1:78.63 g/mL). As in our study, the solvent-liquid ratio had stronger effects than time, with significant time × ratio interaction [54], consistent with SD matrix findings.

**Figure 9:**
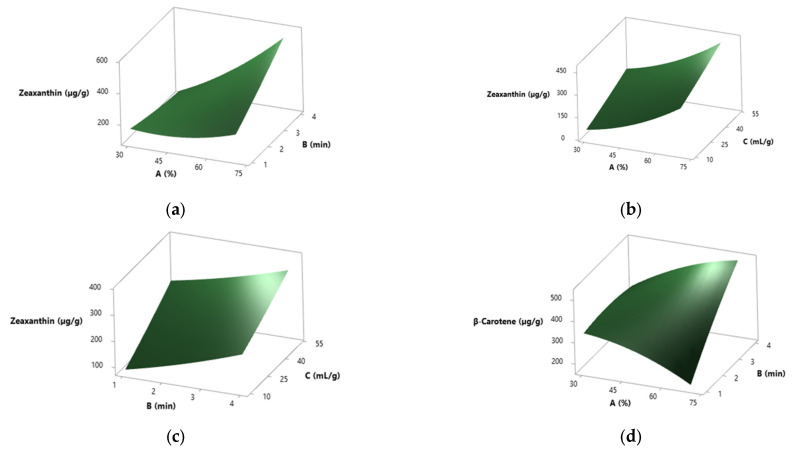

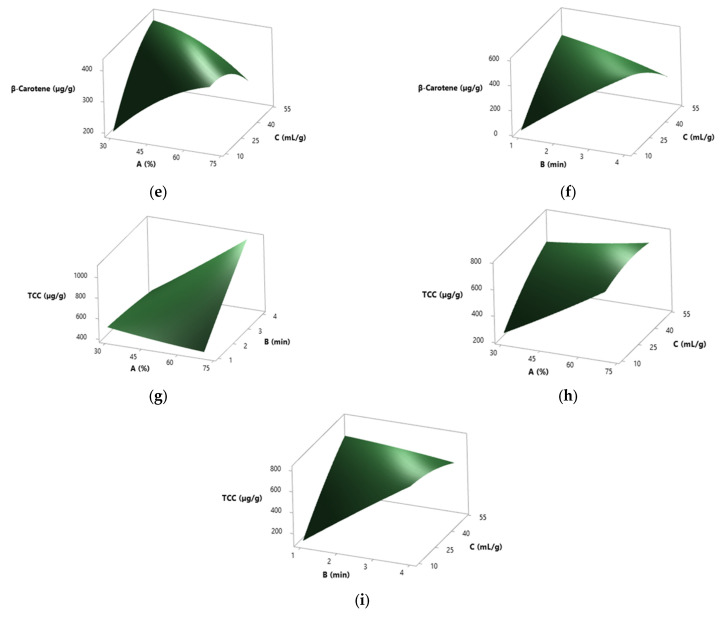
Three-dimensional surface plots showing the interactions between factors in the ultrasound-assisted extraction of zeaxanthin, β-carotene, and total carotenoid content (TCC) from solar-dried *S. platensis*. (**a**–**c**) Interactions for zeaxanthin: (**a**) A vs. B at constant C = 30 mL/g; (**b**) A vs. C at constant B = 2.5 min; (**c**) B vs. C at constant A = 50%; (**d**–**f**) Interactions for β-carotene: (**d**) A vs. B at constant C = 30 mL/g; (**e**) A vs. C at constant B = 2.5 min; (**f**) B vs. C at constant A = 50%; (**g**–**i**) Interactions for total carotenoid content (TCC): (**g**) A vs. B at constant C = 30 mL/g; (**h**) A vs. C at constant B = 2.5 min; (**i**) B vs. C at constant A = 50%. Here, A = Amplitude (%); B = Time (min); C = Solvent-to-solid ratio (mL/g).

For β-carotene, all three independent variables were statistically significant (*p* < 0.001), with the solvent-to-solid ratio showing the strongest effect (*F* = 770.36) (Table S1). Significant two-factor interactions were identified between amplitude and solvent-to-solid ratio (*p* = 0.006), and between time and solvent-to-solid ratio (*p* = 0.023), while the interaction between amplitude and time was not statistically relevant (*p* = 0.079) (Table S1). The interactions between the factors affecting β-carotene extraction are illustrated in the 3D surface plots shown in Figure 9d–f, which depict the significant effects of amplitude, time, and solvent-to-solid ratio as well as their interactions.

The minimum yield for β-carotene was recorded in run 8 (38.1%, 1.6 min, 41.9 mL/g), yielding 442.29 µg/g. In contrast, the highest yield was achieved in run 9 (50%, 2.50 min, 10 mL/g), with a value of 2048.67 µg/g (Table 2). Optimal extraction conditions were defined as 41.7% amplitude, 4 min, and 10 mL/g, yielding 2290.0 µg/g. The actual experimental value obtained under these conditions was 2114.15 µg/g.

In dried *Chlorella* biomass, the UAE (30 kHz) of β-carotene was optimized as sonication time of 7.03 min (5–15 min), ethanol concentration of 62.03% (50–100%), and amplitude of 83.7% (from 20 to 100%). In our study, amplitude and sonication time were both significant factors in the SD matrix. In *Chlorella* biomass, amplitude had the strongest influence, followed by time; ethanol concentration was not significant, although its interaction with amplitude was [55].

A comparable pattern was observed for TCC, where the solvent-to-solid ratio was again the dominant variable, followed by time, and finally amplitude (*p* ≤ 0.001) (Table S1). The interaction between amplitude and solvent-to-solid ratio was significant (*p* < 0.001), while the other interactions did not reach significance (Table S1). The factor interactions influencing TCC are depicted in the 3D surface plots presented in Figure 9g–i, which highlight the dominant effect of the solvent-to-solid ratio and the significant interaction between amplitude and solvent-to-solid ratio. The lowest TCC value was observed in run 8 (38.1%, 1.6 min, 41.9 mL/g), yielding 700.49 µg/g, whereas the highest yield was achieved in run 9 (50%, 2.5 min, 10 mL/g), yielding 2615.44 µg/g (Table 2). The best predicted conditions (41.3% amplitude, 3.73 min, and 10 mL/g) corresponded to a modelled TCC of 2774.5 µg/g, and the experimental yield obtained under these settings was 2805.45 µg/g.

The optimization of the extraction of total pigment yield, assessed spectrophotometrically at 440 nm, from *S. platensis* has already been studied in freeze-dried form in an ultrasonic bath (240 W, 40 kHz) with glucose/glycerol/water-based (1:2:4 molar ratio) NADES. The optimized parameters were temperature (50–70 °C), time (20–40 min), and solvent-to-solid ratio (50–70 mL/mg). Although any of the factors themselves were significant, the interaction between time and solvent-to-solid ratio was significant. The optimized condition was 60 °C, 20 min, 70 mL/mg), giving a total pigment yield of 165.19 mg/g [19]. In contrast to the previous study, in the present work, all three variables, time, amplitude, and solvent-to-solid ratio, were individually significant (*p* < 0.05). Furthermore, the interactions between amplitude and time, as well as amplitude and solvent-to-solid ratio, also showed a significant effect, whereas the time × solvent-to-solid ratio interaction did not.

#### 3.5.2. Solar-Dried *S. platensis*

In the SolD matrix, predictive performance remained high, with R-squared values exceeding 98% for zeaxanthin, β-carotene, and TCC, and statistical significance confirmed for each model. Amplitude, time, and solvent-to-solid ratio significantly influenced the extraction of zeaxanthin and TCC (*p* < 0.001), with particularly notable interaction effects observed in the TCC model (Table S2).

When assessing zeaxanthin, every factor tested was highly significant (*p* < 0.001), with amplitude having the most prominent effect (*F* = 730.23) (Table S2). A significant interaction was observed between amplitude and time (*p* < 0.001), while the remaining interactions did not contribute meaningfully (Table S2). The interactions between factors affecting zeaxanthin extraction in this case are illustrated in the 3D surface plots shown in Figure 9a–c, which emphasize the significant impact of amplitude and its interaction with time.

The poorest yield was registered in run 11 (38.1%, 3.4 min, 18.1 mL/g), with 109.93 µg/g, and the best yield was achieved in run 12 (61.9%, 3.4 min, 18.1 mL/g), reaching 529.87 µg/g (Table 2). The model estimated the optimal point at 70% amplitude, 4 min, and 50 mL/g, with a predicted yield of 629.9 µg/g, while experimental validation gave a slightly lower value of 596.13 µg/g.

Although the optimized conditions for zeaxanthin extraction differed between both matrices with different drying techniques, solid-to-solvent ratio and time were significantly affecting in both matrices, which is in concordance with other studies that have analyzed those parameters in corn [52], marigold flower [53], or other microalgae [54,56,57]. In addition, the interaction between time and amplitude, which has been significant in both matrices, has also been significant in microalgae matrices [56].

In SD *S. platensis,* all interactions were significant, which is also in line with other studies in microalgae, for the other interactions, amplitude and solid-to-solvent ratio interaction [56], and time and solvent-to-solid ratio interaction [54,57].

In freeze-dried *Chlorella saccharophila*, the ultrasound extraction of zeaxanthin (using acetone) was also optimized at 67.38 µL/mg (20–100 µL/mg), 27.82% amplitude (20–60%), 17.9 s pulse (10–50 s), and 13.48 min (5–25 min). Time and solvent-to-solid ratio were key variables, in agreement with our findings, with amplitude contributing mainly through interaction effects [56]. In freeze-dried *Chromochloris zofingiensis*, optimal extraction conditions for canthaxanthin at 50 °C included 49 min (25 to 60 min), 2.3:1 octanoic/decanoic acid ratio (from 1 to 3), and 66.2 mg/mL solid-to-liquid ratio (30 to 70 mg/mL). All individual factors showed a significant influence (similar to the SolD matrix), and both interactions between solvent-to-solid ratio (with acid molar ratio and with time) remained significant [57].

The regression equation for individual and TCC in the SolD matrix, expressed in terms of coded variables, along with the corresponding coefficients of determination (R^2^), is presented in Table 3.

For β-carotene, the key variables with significant effects were time (*p* < 0.001) and solvent-to-solid ratio (*p* = 0.003) (Supplementary Table S2). Although amplitude did not reach significance alone, its role in interaction terms with time and solvent-to-solid ratio was statistically confirmed (*p* < 0.001). Moreover, the interaction between time and solvent-to-solid ratio was also significant (*p* < 0.001) (Supplementary Table S2). The factor interactions influencing β-carotene content are depicted in the 3D surface plots presented in Figure 9d–f, illustrating the significant effects of time and solvent-to-solid ratio as well as the key interactions involving amplitude. The lowest content of β-carotene was found in run 1 (38.1%, 1.6 min, 18.1 mL/g), yielding 215.93 µg/g, whereas run 6 (61.9%, 3.4 min, 18.1 mL/g) produced the highest value, 529.87 µg/g (Figure 5). The model predicted 745.3 µg/g as the maximum at 70% amplitude, 4 min, and 10 mL/g. Experimentally, this condition yielded 693.32 µg/g.

In both matrices studied, time and solvent-to-solid ratio were significant factors in the extraction of β-carotene, similarly to what was observed for zeaxanthin. This aligns with previous findings in *Chlorella saccharophila*, where both variables influenced the extraction efficiency [55]. Additionally, the solvent-to-solid ratio has been identified as a key parameter in *Chlorella vulgaris* [52,58], while extraction time was also reported as relevant in other studies using *Chlorella* biomass [55]. Notably, the interactions between amplitude and solvent-to-solid ratio, as well as between time and solvent-to-solid ratio, were significant in both matrices. The latter interaction has also been reported as relevant in other microalgae systems [58].

Our results agree with those of Singh et al. [56], who found similar behaviour in freeze-dried *C. saccharophila*. The ultrasound extraction of β-carotene (using acetone) was also optimized (67.38 µL/mg of solvent-to-solid ratio, 27.82% of ultrasound power, 17.9 s pulse, and 13.48 min of extraction time). The solvent-to-solid ratio was the most influential, followed by time and power, although all individual factors remained significant (*p* < 0.05) as in both matrices of *S. platensis*. Interactions between the solvent-to-solid ratio and pulse resulted in significant changes in β-carotene [56].

In *C. vulgaris* lyophilized powder with an ultrasonic bath (40 kHz), optimized conditions (85 min (15–155 min), 94% ethanol (35–95%), 66 °C (15–75 °C), 103 mL/g (30–150 mL/g)) showed that solvent concentration and temperature were key variables [41]. Solvent concentration also showed a significant effect in both *S. platensis* matrices for β-carotene extraction.

As for TCC, all main factors demonstrated highly significant effects (*p* < 0.001), and all interaction terms were also statistically relevant (*p* < 0.001) (Supplementary Table S2). The interaction between time and solvent-to-solid ratio is the most influential (*F* = 238.23), followed by amplitude and time (*F* = 213.75) (Supplementary Table S2). The interactions between factors affecting TCC are visualized in the 3D surface plots shown in Figure 9g–i, highlighting the significance of all main factors and their interactions, particularly between time and solvent-to-solid ratio. The least favourable condition was found in run 1 (38.1%, 1.6 min, 18.1 mL/g), giving 326.47 µg/g, while run 6 (61.9%, 3.4 min, 18.1 mL/g) resulted in the highest TCC yield (869.36 µg/g) (Figure 5). The same optimal parameters defined for β-carotene also applied to TCC, with a predicted value of reaching 1216.3 µg/g, and a corresponding experimental result of 1205.48 µg/g.

As mentioned in the previous section, the optimization of total pigment yield extraction, assessed spectrophotometrically at 440 nm, from *S. platensis* (freeze-dried) was previously studied using an ultrasonic bath (240 W, 40 kHz) and a glucose/glycerol/water-based NADES (1:2:4 molar ratio). The optimized parameters included temperature (50–70 °C), extraction time (20–40 min), and solvent-to-solid ratio (50–70 mL/mg). Although none of the individual factors were statistically significant, the interaction between time and solvent-to-solid ratio, as also observed in the SolD matrix, showed a significant effect. The optimal conditions (60 °C, 20 min, 70 mL/mg) yielded 165.19 mg/g of total pigments [19].

TCC behaved as an integrative variable, reflecting both individual carotenoid yields and overall matrix accessibility. In the SD matrix, the solvent-to-solid ratio was again the dominant factor, followed by time and by amplitude. Interaction terms were less determinant than in SolD, being only amplitude and solvent-to-solid ratio significant (*p* < 0.001) (Figure 8g–i). Conversely, in the SolD matrix, all interaction terms were significant (*p* < 0.001), particularly time × solvent-to-solid ratio (*F* = 238.23) (Figure 9g–i). This indicates that more aggressive extraction conditions were needed to compensate for reduced carotenoid availability in the solar-dried material. With optimized extraction conditions for TCC (including zeaxanthin and β-carotene contents) from SD *S. platensis*, a yield of 2805.45 µg/g and an increase of 3.60-fold over the initial non-optimized value of 779.07 µg/g were found. In the SolD matrix, the increase was 4.43-fold, with optimized conditions yielding 1205.48 µg/g versus 285.04 µg/g under non-optimized conditions.

The optimized TCC in the SD matrix (2805.45 µg/g) far surpassed that of the SolD matrix (1205.48 µg/g), being 2.33 times higher. This difference is consistent with previous studies reporting that rapid drying techniques better retain the integrity of thermolabile bioactive compounds [59]. Spray drying is a rapid and efficient dehydration method that offers better control over temperature and moisture removal, thereby minimizing thermal degradation and microbial growth. It also produces a homogeneous fine powder with consistent quality, which is highly desirable for food or nutraceutical applications. In contrast, solar drying, despite being the most accessible and cost-effective method, exhibits substantial limitations. These include a strong dependency on weather conditions, longer drying times, and less control over processing parameters, all of which can lead to biomass degradation, color alteration, and increased microbial load. As reported in the literature, solar drying may result in inconsistent product quality and is not recommended for applications where human consumption or bioactive preservation is a priority [60,61]. Nonetheless, the moderate TCC achieved under optimized conditions for the SolD matrix demonstrates that, while less effective, solar drying can still be a viable option when economic constraints or energy limitations are present.

#### 3.5.3. Solvent Effect

The use of different solvents, 2-MeTHF for SD and ethanol for SolD, may have contributed to differences in extraction efficiency. 2-MeTHF has lower polarity (1.38 D) and viscosity (0.60 cP) than ethanol (1.66 D, 1.10 cP), which likely enhanced β-carotene solubilization and diffusion in SD samples [62,63]. Interestingly, in the SolD matrix, the β-carotene content was only 1.16 times higher than zeaxanthin, while in the SD matrix, β-carotene exceeded zeaxanthin content by 2.71-fold.

In contrast, ethanol may have better penetrated the more compact structure of SolD biomass, improving zeaxanthin recovery despite its lower affinity for hydrophobic compounds. These solvent–matrix interactions can significantly affect extraction efficiency and compound selectivity, as the optimal solvent depends on the complexity of the matrix and the polarity of the carotenoids [16,64].

It is worth mentioning that the integrated approach of combining COSMO-SAC computational predictions with experimental validation and RSM optimization for both *Spirulina* drying matrices offers a robust and innovative framework for future bioactive compound extraction studies. This methodology allowed us to identify optimal conditions for each specific *Spirulina* form, demonstrating superior recovery efficiencies that surpass what could be achieved with single-solvent or non-optimized methods.

## 4. Conclusions

This study demonstrated the significant impact of drying methods and solvent selection on carotenoid extraction efficiency from *S. platensis*. SD *S. platensis* consistently showed higher carotenoid extraction yields for most solvents compared to the SolD sample. This result is in line with the particle size analysis, which indicated a lower degree of aggregation in the SD sample. The carotenoid composition was also influenced by the drying method, with a higher proportion of zeaxanthin relative to β-carotene observed in the SD sample than in the SolD sample. Regarding solvent efficacy, it was found to depend not only on the drying method used but also on the specific carotenoid extracted. Acetone was the most effective solvent for extracting both zeaxanthin and β-carotene from SD *S. platensis*, and for extracting β-carotene from the SolD sample. For zeaxanthin extraction from the SolD sample, methanol and ethanol yielded the best results.

Considering the emerging biosolvents ethyl lactate and 2-MeTHF, based on the COSMO-SAC analysis and the quantification of individual and total carotenoids, the former was the least effective solvent for carotenoid extraction in both SD and SolD samples. In contrast, 2-MeTHF not only showed the best COSMO-SAC-predicted affinity for both zeaxanthin and β-carotene, but also ranked as the second most effective solvent for their extraction from SD *S. platensis*. These results highlight its potential as a sustainable and efficient alternative to traditional solvents.

Considering the strong agreement between COSMO-SAC predictions and experimental outcomes, this study reinforces the value of using computational models to guide solvent selection for the targeted extraction of bioactive compounds from food matrices.

The development of RSM-optimized models for ultrasound-assisted carotenoid extraction using 2-MeTHF (SD) and ethanol (SolD) proved highly successful, achieving excellent predictive capabilities (R^2^ > 98%) for key carotenoids (zeaxanthin and β-carotene), as well as TCC. This research effectively identified optimal extraction parameters of 10 mL/g solvent-to-solid ratio and an approximate 4 min ultrasound duration for maximizing TCC from *S. platensis* in both solvent systems. A crucial finding was the divergence in optimal ultrasound amplitude between the two solvents (70% for SolD and 41.3% for SD), underscoring the critical need for sample- and solvent-specific optimization strategies. Ultimately, the significant four-fold increase in TCC yield under optimized conditions, compared to non-optimized approaches, emphatically validates the efficacy of the developed models and the importance of tailored extraction protocols for efficient carotenoid recovery.

This study provides novel insights into the ultrasound-assisted extraction of carotenoids from *S. platensis*, demonstrating that drying methods critically influence extraction profiles. We further establish the promising role of 2-mTHF as an efficient and sustainable biosolvent, validated by computational and experimental data, offering a greener alternative for the industry. The optimized extraction protocols for both spray-dried and solar-dried biomass represent a significant step towards scalable and efficient carotenoid production from *Spirulina*.

Furthermore, our results demonstrate that combining computational predictions with experimental validation is an effective approach to develop sustainable and efficient extraction methods for health-promoting compounds from *S. platensis*, a rich natural source of carotenoids, emphasizing the benefit of integrating both tools to optimize recovery from complex natural sources.

Finally, as for potential applications, carotenoids extracted from *S. platensis* are versatile compounds with proven health benefits, including the reduction in cancer risk, cardiovascular diseases, and eye and skin conditions, in addition to their crucial role in combating vitamin A deficiency [65]. Therefore, our findings are highly relevant for the application of these carotenoid-rich extracts in the formulation of functional foods, nutraceuticals, food supplements, and nutricosmetics. The efficacy of green biosolvents, especially those with regulatory approval, underscores the potential of our method for the development of more sustainable and safer extraction processes in the industry, opening new avenues for the valorization of *S. platensis.*

## Figures and Tables

**Table 1:** Central composite design (uncoded) applied to optimize carotenoid extraction from SD and SolD *Spirulina platensis* (A: Amplitude; B: Time; C: Solvent-to-solid ratio).

Run	A (%)	B (min)	C (mL/g)
1	38.1	1.6	18.1
2	50.0	2.5	30.0
3	50.0	1.0	30.0
4	61.9	1.6	18.1
5	50.0	2.5	30.0
6	61.9	3.4	18.1
7	50.0	2.5	30.0
8	38.1	1.6	41.9
9	50.0	2.5	10.0
10	70.0	2.5	30.0
11	38.1	3.4	18.1
12	61.9	3.4	41.9
13	38.1	3.4	41.9
14	30.0	2.5	30.0
15	50.0	2.5	30.0
16	61.9	1.6	41.9
17	50.0	2.5	30.0
18	50.0	4.0	30.0
19	50.0	2.5	30.0
20	50.0	2.5	50.0

**Table 2:** Central composite design (uncoded) applied to optimize carotenoid extraction and experimental results of zeaxanthin, β-carotene, and total carotenoid content (TCC) (µg/g) from spray-dried (SD) and solar-dried (SolD) *S. platensis*. A: Amplitude (%); B: Time (min); C: Solvent-to-solid ratio (mL/g).

Run	A	B	C	SD			SolD		
				Zeaxanthin	β-Carotene	TCC	Zeaxanthin	β-Carotene	TCC
1	38.1	1.6	18.1	670.24	1249.27	1919.51	110.54	215.93	326.47
2	50.0	2.5	30.0	602.89	1106.14	1709.02	230.55	381.37	611.92
3	50.0	1.0	30.0	363.24	866.23	1229.47	166.33	275.30	441.63
4	61.9	1.6	18.1	347.50	1338.45	1685.95	191.04	230.41	421.45
5	50.0	2.5	30.0	631.50	1140.87	1772.37	212.52	385.44	597.96
6	61.9	3.4	18.1	532.55	1627.99	2160.54	339.49	529.87	869.36
7	50.0	2.5	30.0	571.99	1106.14	1678.13	219.64	394.19	613.83
8	38.1	1.6	41.9	258.20	442.29	700.49	186.05	455.35	641.40
9	50.0	2.5	10.0	566.78	2048.67	2615.44	142.47	337.68	480.15
10	70.0	2.5	30.0	492.65	1094.56	1587.20	380.94	357.12	738.06
11	38.1	3.4	18.1	588.36	1644.27	2232.63	109.93	388.14	498.07
12	61.9	3.4	41.9	557.96	876.77	1434.73	419.22	375.73	794.95
13	38.1	3.4	41.9	277.25	676.60	953.85	194.73	358.13	552.86
14	30.0	2.5	30.0	486.35	832.88	1319.23	136.85	352.46	489.31
15	50.0	2.5	30.0	602.51	1048.45	1650.95	204.16	405.48	609.64
16	61.9	1.6	41.9	329.58	829.00	1158.58	293.21	344.67	637.88
17	50.0	2.5	30.0	605.65	1219.43	1825.09	210.31	382.34	592.65
18	50.0	4.0	30.0	559.40	1279.41	1838.81	290.51	459.98	750.49
19	50.0	2.5	30.0	588.01	1106.14	1694.15	218.44	387.33	605.77
20	50.0	2.5	50.0	275.76	631.32	907.07	308.43	352.31	660.74

**Table 3:** Regression equations (in coded variables) for zeaxanthin, β-carotene and total carotenoid content (TCC) in spray-dried (SD) and solar-dried (SolD) *S. platensis*, obtained from the RSM study.

Response		Regression Model (Coded Variables)	R^2^
SD	Zeaxanthin	980 − 5.25 A + 38.0 B − 16.78 C − 0.2825 A×A − 62.75 B × B − 0.4531 C × C + 5.613 A × B + 0.6458 A × C + 1.700 B × C	0.9905
	β-Carotene	443 + 47.1 A + 612 B − 73.45 C − 0.4537 A × A − 32.2 B × B + 0.4870 C × C − 3.44 A × B + 0.454 A × C − 4.74 B × C	0.9896
	TCC	1423 + 41.9 A + 650 B − 90.2 C − 0.736 A × A − 94.9 B × B + 0.034 C × C + 2.17 A × B + 1.100 A × C − 3.04 B × C	0.9915
SolD	Zeaxanthin	354.3 − 11.43 A − 128.6 B + 2.58 C + 0.0949 A × A + 3.33 B × B + 0.0113 C × C + 3.139 A × B + 0.0191 A × C − 0.155 B × C	0.9921
	β-Carotene	−564 + 6.89 A + 135.4 B + 33.95 C − 0.0737 A × A − 7.40 B × B − 0.0982 C × C + 3.011 A × B − 0.2203 A × C − 6.338 B × C	0.9819
	TCC	−210 − 4.54 A + 6.7 B + 36.53 C + 0.0212 A × A − 4.07 B × B − 0.0869 C × C + 6.151 A × B − 0.2013 A × C − 6.493 B × C	0.9952

A: Amplitude (%); B: Time (min); C: Solvent-to-solid ratio (mL/g).

## Data Availability

Data are contained within the article and Supplementary Materials.

## Supplementary Materials

The following supporting information can be downloaded at: https://www.mdpi.com/article/10.3390/molecules30193881/s1, Figure S1: Particle size distribution by intensity of SolD (a) and SD (b), where the X-axis represents the particle size (d.nm) on a logarithmic scale, and the Y-axis represents the intensity percentage.; Table S1: ANOVA results for the models of individual carotenoids (zeaxanthin and β-carotene) and total carotenoid content (TCC) (µg/g) in spray-dried (SD) *Spirulina platensis*. A: Amplitude (%); B: Time (min); C: Solvent-to-solid ratio (mL/g). DF: Degrees of Freedom; Adj SS: Adjusted Sum of Squares; Adj MS: Adjusted Mean Square; Table S2: ANOVA results for the models of individual carotenoids (zeaxanthin and β-carotene) and total carotenoid content (TCC) (µg/g) in solar dried (SolD) *Spirulina platensis*. A: Amplitude (%); B: Time (min); C: Solvent-to-solid ratio (mL/g). DF: Degrees of Freedom; Adj SS: Adjusted Sum of Squares; Adj MS: Adjusted Mean Square.
