# Supplementary material for: Ultrasound-Assisted Extraction of Spirulina platensis Carotenoids: Effect of Drying Methods and Performance of the Emerging Biosolvents 2-Methyltetrahydrofuran and Ethyl Lactate
Source: Molecules. 2025 Sep 25;30(19):3881. doi: 10.3390/molecules30193881 (PMC12525988; doi:10.3390/molecules30193881)
Supplement: Supplementary file 1 [file molecules-30-03881-s001.zip › Figure S1 update 15.9.pptx]

## Slide 1
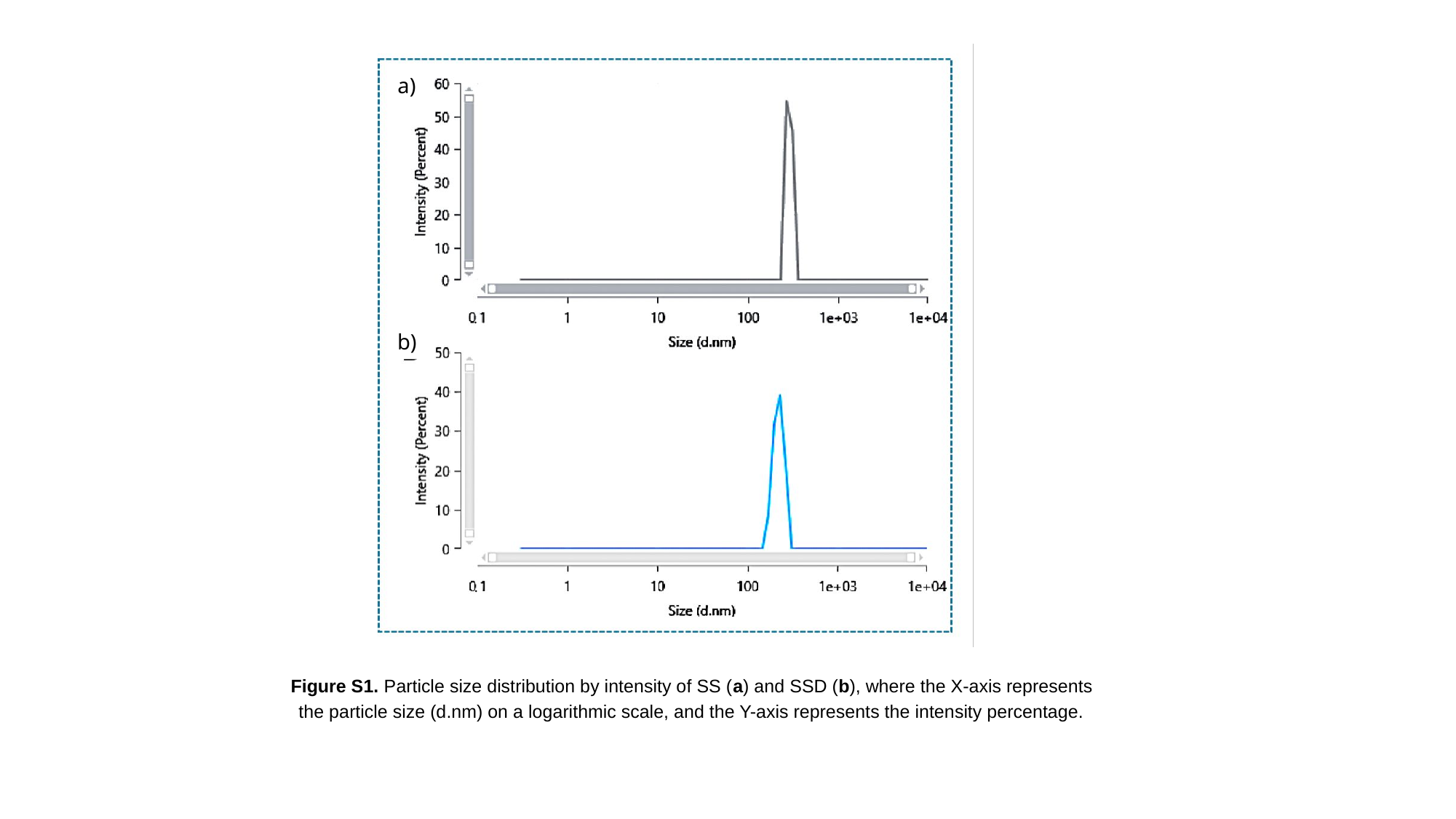

a)
b)
.
.
Figure S1. Particle size distribution by intensity of SS (a) and SSD (b), where the X-axis represents the particle size (d.nm) on a logarithmic scale, and the Y-axis represents the intensity percentage.
