# Supplementary material for: Ultrasound-Assisted Extraction of Spirulina platensis Carotenoids: Effect of Drying Methods and Performance of the Emerging Biosolvents 2-Methyltetrahydrofuran and Ethyl Lactate
Source: Molecules. 2025 Sep 25;30(19):3881. doi: 10.3390/molecules30193881 (PMC12525988; doi:10.3390/molecules30193881)
Supplement: Supplementary file 1 [file molecules-30-03881-s001.zip › Table S1.pdf]

Table S1. ANOVA results for the models of individual carotenoids (zeaxanthin and β-carotene) and total carotenoid content (TCC) (μg/g) in spray-dried (SD) Spirulina platensis. A: Amplitude (%); B: Time (min); C: Solvent-to-solid ratio (mL/g). DF: Degrees of Freedom; Adj SS: Adjusted Sum of Squares; Adj MS: Adjusted Mean Square.

SD *Spirulina platensis*					
**Source**	**DF**	**Adj SS**	**Adj MS**	***F*-Value**	***P*-Value**
**Zeaxanthin**					
Model	9	337630	37514	116.05	0
Linear	3	140265	46755	144.63	0
A	1	18	18	0.06	0.816
B	1	33910	33910	104.9	0
C	1	106337	106337	328.94	0
Square	3	99679	33226	102.78	0
A*A	1	23002	23002	71.15	0
B*B	1	35905	35905	111.07	0
C*C	1	59167	59167	183.03	0
2-Way Interaction	3	97686	32562	100.73	0
A*B	1	28353	28353	87.71	0
A*C	1	66731	66731	206.42	0
B*C	1	2601	2601	8.05	0.018
Error	10	3233	323		
Lack-of-Fit	5	1267	253	0.64	0.679
Pure Error	5	1966	393		
Total	19	340863			
R^2			0.9905		
Adjusted R^2			0.9820		
**β-Carotene**					
Model	9	2656567	295174	105.75	0
Linear	3	2440986	813662	291.5	0
A	1	88579	88579	31.73	0
B	1	202138	202138	72.42	0
C	1	2150269	2150269	770.36	0
Square	3	151655	50552	18.11	0
A*A	1	59320	59320	21.25	0.001
B*B	1	9433	9433	3.38	0.096
C*C	1	68363	68363	24.49	0.001
2-Way Interaction	3	63926	21309	7.63	0.006
A*B	1	10658	10658	3.82	0.079
A*C	1	33022	33022	11.83	0.006
B*C	1	20246	20246	7.25	0.023
Error	10	27913	2791		
Lack-of-Fit	5	11902	2380	0.74	0.624
Pure Error	5	16011	3202		
Total	19	2684480			
R^2			0.9896		
Adjusted R^2			0.9802		
**TCC**					
Model	9	4131332	459037	129.21	0
Linear	3	3700631	1233544	347.23	0
A	1	86041	86041	24.22	0.001
B	1	401632	401632	113.06	0
C	1	3212958	3212958	904.41	0

Square	3	224485	74828	21.06	0
A*A	1	156200	156200	43.97	0
B*B	1	82145	82145	23.12	0.001
C*C	1	332	332	0.09	0.766
2-Way Interaction	3	206216	68739	19.35	0
A*B	1	4244	4244	1.19	0.3
A*C	1	193638	193638	54.51	0
B*C	1	8334	8334	2.35	0.157
Error	10	35525	3553		
Lack-of-Fit	5	14446	2889	0.69	0.656
Pure Error	5	21080	4216		
Total	19	4166857			
R^2			0.9915		
Adjusted R^2			0.9838		
