# Supplementary material for: Ultrasound-Assisted Extraction of Spirulina platensis Carotenoids: Effect of Drying Methods and Performance of the Emerging Biosolvents 2-Methyltetrahydrofuran and Ethyl Lactate
Source: Molecules. 2025 Sep 25;30(19):3881. doi: 10.3390/molecules30193881 (PMC12525988; doi:10.3390/molecules30193881)
Supplement: Supplementary file 1 [file molecules-30-03881-s001.zip › Table S2.pdf]

Table S2. ANOVA results for the models of individual carotenoids (zeaxanthin and β-carotene) and total carotenoid content (TCC) (µg/g) in solar dried (SolD) *Spirulina platensis.* A: Amplitude (%); B: Time (min); C: Solvent-to-solid ratio (mL/g). DF: Degrees of Freedom; Adj SS: Adjusted Sum of Squares; Adj MS: Adjusted Mean Square.

SolD *Spirulina platensis*

**Source**	**DF**	**Adj SS**	**Adj MS**	***F*-Value**	***P*-Value**
**Zeaxanthin**					
Model	9	138588	15398,6	138.7	0
Linear	3	127016	42338,8	381.36	0
A	1	81070	81070	730.23	0
B	1	17679	17678,9	159.24	0
C	1	28267	28267,4	254.61	0
Square	3	2621	873,8	7.87	0.005
A*A	1	2596	2595,8	23.38	0.001
B*B	1	101	101	0.91	0.363
C*C	1	37	36,8	0.33	0.578
2-Way Interaction	3	8950	2983,3	26.87	0
A*B	1	8870	8870	79.9	0
A*C	1	58	58,3	0.52	0.485
B*C	1	22	21,6	0.19	0.669
Error	10	1110	111		
Lack-of-Fit	5	695	139	1.67	0.293
Pure Error	5	415	83,1		
Total	19	139698			
R^2			0.9921		
Adjusted R^2			0.9849		
**β-Carotene**					
Model	9	96938,4	10770,9	60.31	0
Linear	3	40677,9	13559,3	75.92	0
A	1	368,9	368,9	2.07	0.181
B	1	37549,3	37549,3	210.25	0
C	1	2759,7	2759,7	15.45	0.003
Square	3	4171,8	1390,6	7.79	0.006
A*A	1	1566,9	1566,9	8.77	0.014
B*B	1	498,9	498,9	2.79	0.126
C*C	1	2780,7	2780,7	15.57	0.003
2-Way Interaction	3	52088,7	17362,9	97.22	0
A*B	1	8161,7	8161,7	45.7	0
A*C	1	7768,5	7768,5	43.5	0
B*C	1	36158,6	36158,6	202.47	0
Error	10	1785,9	178,6		
Lack-of-Fit	5	1370	274	3.29	0.108
Pure Error	5	415,9	83,2		
Total	19	98724,3			
R^2			0.9819		
Adjusted R^2			0.9656		
**TCC**					
Model	9	328824	36536	229.37	0
Linear	3	247826	82609	518.6	0
A	1	92376	92376	579.92	0
B	1	106758	106758	670.2	0
C	1	48692	48692	305.68	0
Square	3	2521	840	5.27	0.019

A*A	1	129	129	0.81	0.389
B*B	1	151	151	0.95	0.353
C*C	1	2178	2178	13.67	0.004
2-Way Interaction	3	78478	26159	164.22	0
A*B	1	34049	34049	213.75	0
A*C	1	6481	6481	40.69	0
B*C	1	37948	37948	238.23	0
Error	10	1593	159		
Lack-of-Fit	5	1244	249	3.56	0.095
Pure Error	5	349	70		
Total	19	330417			
R^2				0.9952	
Adjusted R^2				0.9908	
